# Consistent condom use with regular, paying, and casual male partners and associated factors among men who have sex with men in Tamil Nadu, India: findings from an assessment of a large-scale HIV prevention program

**DOI:** 10.1186/1471-2458-13-827

**Published:** 2013-09-11

**Authors:** Shreena Ramanathan, Venkatesan Chakrapani, Lakshmi Ramakrishnan, Prabuddhagopal Goswami, Diwakar Yadav, Thilakavathi Subramanian, Bitra George, Ramesh Paranjape

**Affiliations:** 1 FHI 360 India, H-5 (Ground Floor), Green Park Extension, New Delhi 110016, India; 2 Centre for Sexuality and Health Research and Policy (C-SHaRP), 38 (Old No.167), Rangarajapuram Main Road, Kodambakkam, Chennai 600 024, India; 3 National Institute of Epidemiology, Second Main Road, TNHB, Ayapakkam, Chennai 600 077, India; 4 National AIDS Research Institute, T 71-1A/2, M.I.D.C., Telco Road, Bhosari, Pune 411 026, India

**Keywords:** Men who have sex with men, MSM, Condom use, Male sexual partners, Sexual risk, Tamil Nadu, India

## Abstract

**Background:**

Men who have sex with men (MSM) are a marginalized population at high risk for HIV infection. Promoting consistent condom use (CCU) during anal sex is a key risk reduction strategy for HIV prevention among MSM. To inform effective HIV prevention interventions, we examined the factors associated with CCU among MSM with their regular, paying, and casual partners, as well as with all three types of partners combined.

**Methods:**

Data for this analysis were from a large-scale bio-behavioural survey conducted during 2009–2010 in Tamil Nadu, India. MSM aged 18 years or older were recruited for the survey using time-location cluster sampling at cruising sites in four districts of Tamil Nadu. Binary logistic regression analyses were conducted to assess the association of CCU with selected socio-demographic characteristics and other contextual factors.

**Results:**

Among 1618 MSM interviewed, CCU during anal sex with regular, paying, and a casual male partner was 45.3%, 50.8% and 57.9%, respectively. CCU with all three types of partners combined was 52.6%. Characteristics associated with increased odds for CCU with MSM having all three types of partners combined were frequent receptive anal sex acts with regular partners (adjusted odds ratio [AOR] 2.17, 95% confidence interval [CI] 1.01-4.65), fewer number of casual partners (AOR 3.41, 95% CI 1.50-7.73) and membership in a community-based organization (CBO) for MSM (AOR 3.54, 95% CI 1.62-7.74). CCU with regular partners was associated with membership in a CBO (AOR 1.96, 95% CI 1.23-3.11), whereas CCU with paying, and casual male partners was associated with perceived higher risk of acquiring HIV (AOR 1.92, 95% CI 1.22-3.01) and exposure to any HIV prevention intervention (AOR 3.62, 95% CI 1.31-10.0), respectively. Being aged 26 years or older, being in debt, and alcohol use were factors associated with inconsistent condom use across partner types.

**Conclusion:**

HIV interventions among MSM need to promote CCU with all types (regular, paying, and causal) of male partners, and need to reach MSM across all age groups. In addition to enhancing interventions that focus on individual level risk reduction, it is important to undertake structural interventions that promote social acceptance of same-sex sexuality and address contextual barriers to condom use such as alcohol use.

## Background

Men who have sex with men (MSM) are a vulnerable population at high risk for HIV, accounting for 5 to 10% of HIV-infected populations globally [1-3]. The prevalence of HIV among MSM in India is showing an increasing trend, including newly recognized populations of MSM in the low HIV prevalence states [4]. According to the National AIDS Control Organization (NACO) of India, the national average HIV prevalence among MSM was 7.3% in 2009, twenty times higher than that among the general population (0.31%) [4,5]. The Indian state of Tamil Nadu has historically been one among the six highest HIV prevalence states, [6,7] with HIV prevalence among MSM increasing from 4.2% to 6.6% between 2003 and 2007 [4].

Despite a long history of existence of people with diverse sexualities in India [8,9] open discussions about same-sex sexuality in the public domain are still limited. With long-standing patriarchal norms in most sections of the Indian society, irrespective of one’s sexual orientation or behavior, males are expected to perform the traditional masculine roles, including getting married to a woman and sustaining their family lineage. Thus, having an identity based on one’s sexuality is alien to most of the Indians, although this situation is changing now [8]. Furthermore, consensual same-sex relationships in India were criminalised until July 2009 and a case filed against this judgement is currently in Supreme Court [10]. The socio-cultural and legal systems, thus, put forth a hostile environment which propagates stigma, shame and discrimination that shape the lived experiences of MSM in India. Self-stigma, often secondary to lack of social acceptance, leads to depression and substance abuse which in turn may be associated with risky sexual practices [11-13]. MSM are also known to have multiple male sexual partners and inconsistent condom use has been reported to be high [14-18]. Having multiple partners and inconsistent condom use are strongly associated with a high risk of transmission and acquisition of HIV and other sexually transmitted infections (STIs) [2,7,19]. Promoting consistent condom use during anal sex is a key risk reduction strategy for HIV prevention among MSM [20-22]. While awareness of condoms as a method for prevention of HIV is high among MSM in India, consistent condom use is low and has been found to vary according to the type of partner [23-25]. In NACO’s Behavioural Surveillance Survey (2006), consistent condom use among MSM ranged from 6.6% in Uttar Pradesh to 65.2% in Goa with commercial male partners, and from 4.8% in Uttar Pradesh to 79% in Mumbai with non-commercial male partners [23]. Findings from the Integrated Behavioral and Biological Assessment (IBBA) conducted with self-identified MSM (in 2005–2006) across four southern states showed that consistent condom use was overall low: 29% with non-commercial non-regular male partners and 49% with regular male partners [24].

Studies from India have found several factors that were associated with inconsistent condom use specified as barriers [26] or contextual factors [27]: non-availability of condoms, trust and intimacy, partner’s objection to condom use, condom use seen as partner’s responsibility, multiple encounters in cruising sites, dislike or dissatisfaction with condoms [26,27], middle socioeconomic class and increased frequency of sex [28]. Descriptive studies indicate that consistent condom use rates vary according to partner types [15,24,27], but studies examining factors associated with condom use have not specifically examined factors associated with consistent condom use with different types of male partners. Given the concurrent sexual partnerships among MSM with different types of male partners [29,30], it is important to understand the factors associated with consistent condom use with each type of male partner (regular, casual, and paying) as well as all three types of partners combined. To fill this crucial gap, in this paper, we focus on examining predictors of consistent condom use to inform HIV prevention interventions targeted towards high-risk MSM given the reported differences in consistent condom use with the type of male partners. In the current context of unrelenting HIV epidemic among MSM in India [31], to strengthen the effectiveness of existing HIV interventions, it is important to understand the factors that facilitate MSM to consistently use condoms with different types of partners (regular, casual and paying).

## Methods

### Data source

Two rounds of IBBA were conducted as part of evaluation of a large-scale HIV prevention program, in 2005–2006 (Round 1) and 2009–2010 (Round 2). The IBBA was intended to be a repeat cross-sectional survey and differed from the usual second generation surveillance model as it collected both behavioral data and biological specimens from same sample of individuals. Survey groups included female sex workers and their clients, MSM, injecting drug users and long distance truck drivers. Conventional cluster and time-location sampling were the methods used for selection of respondents. The detailed IBBA methodology has been documented elsewhere [32-34].

The data for the current analyses were from the second round of IBBA, which was conducted in four districts of Tamil Nadu (Chennai, Madurai, Coimbatore and Salem). Men aged 18 years or older who had anal sex with other men in exchange for cash or in kind during the month prior to the survey were recruited from cruising sites (such as bus stops, railway stations and public toilets) using time-location cluster sampling. Written informed consent was obtained from all respondents and data on demographics, condom use, sexual partners, STIs, and exposure to program interventions were gathered using structured questionnaires during face-to-face interviews along with biological specimens that were tested for HIV and other STIs. The study was approved by the Protection of Human Subjects Committee of FHI 360, and local ethics committees of the implementing Indian Council of Medical Research Institutes (National AIDS Research Institute, Pune, and National Institute of Epidemiology, Chennai).

In this analysis, we focused on reported consistency of condom use among MSM with their regular, paying and casual male partners. For the survey purposes, the operational definitions for the types of male partners are: 1) Regular partner - main partner to whom the participant feels committed, such as spouse, lover or boyfriend; 2) Paying partner - person who have paid the participant in cash or kind for sex; 3) Casual partner - a stranger, friend or acquaintance with whom the participant had sex and does not consider as a regular or paying partner; 4) All three types of partners combined-those MSM having regular, paying, and casual male partners.

### Measures

#### ***Dependent variables***

The primary outcome indicator was self-reported consistency in condom use during anal receptive and/or insertive sex with different (regular, paying, and casual) male partners and all three types of partners combined. MSM who reported using condoms every time during anal sex were considered to be consistent condom users, and those who reported using condoms most of the time, sometimes, and never were considered as inconsistent condom users. Given that a single act of unprotected sex may result in exposure to HIV or other STIs, those reporting condom use most of the time were also grouped with inconsistent users. The condom use variables were dichotomized as consistent versus inconsistent for the current analysis.

#### ***Independent variables***

The following independent variables were examined to assess their relationship with reported consistency in condom use.

#### ***Socio-demographic variables***

Demographic variables included: age in completed years, education (highest grade completed), primary occupation (unemployed, student, self-employed/ professional, non-agricultural labor, business/trade, service, agricultural labor, masseur, sex work, transport workers; only one response allowed), marital status (ever married/never married), self-reported sexual identity (such as kothi, panthi, bisexual, double-decker) and in-debt (Yes/No). Kothis are self-identified MSM who are generally feminine and receptive partners during anal sex, and panthis are those who are masculine and insertive partners. Here ‘bisexual’ refers to the self-reported identity of some proportion of respondents. The term ‘double-decker’ (also called ‘AC/DC’ in some study sites) refers to those men who are both insertive and receptive partners in anal sex.

#### ***Alcohol and drug use***

Substance use was measured separately for alcohol and drug use. Respondents were asked how often they consumed alcoholic drinks in the past month (every day, at least once a week, less than once a week, not in the past month, never) and if they have ever used drugs such as marijuana, heroin, and amphetamines (Yes/No).

#### ***Frequency and number of sex acts/male partners***

We assessed frequency of anal receptive sex with regular male partner (daily, more than twice a week, twice a week, once a week, less than once a week and never), number of sex acts with paying male partners in the past week, and number of casual male partners in the past month.

#### ***Risk perception, knowledge of STIs, membership in a community-based organization for MSM and HIV prevention program exposure***

These variables included self-risk perception of HIV risk (Yes/No), knowledge of any three or more symptoms of STIs (such as genital or anal ulcer, discharge from rectum, pain during defecation, burning sensation upon urination, urethral discharge, swelling in groin), membership in a community-based organization of MSM (a formal group comprised of and managed by MSM - Yes/No) and a composite indicator on program exposure was created to assess the influence of HIV prevention program on condom use. The indicator was based on respondents having received any one of the three core program services (peer outreach, condom distribution and STI clinic) from any non-governmental agency working with MSM in the year prior to the survey. Those who reported receiving any one of the three services were considered to be exposed (coded as ‘yes’) and the remaining as unexposed (coded as ‘no’).

### Data analyses

The data from all four districts were merged for the analysis and appropriate weights were applied. At the district level, weighting was based on the clustering effect of the sample. At the aggregate level, standardized weights were calculated combining the four districts. Additional details on the weighing process followed in IBBA are available in the IBBA operational manual, a guide for conducting IBBA among high-risk population groups [34]. ‘Missing/don’t know’ cases were excluded from the analysis. Univariate, bivariate and multivariate analyses were conducted using Stata version 11® (Stata Corporation, College Station, TX). Univariate analysis was conducted to describe the sociodemographic and other relevant characteristics of the population. Bivariate analysis was conducted to assess the prevalence of outcome measure (consistent condom use) among MSM by socio-demographic and other contextual factors. Multiple logistic regression models were fitted to examine the correlates of consistent condom use according to different partner types and all three types of partners combined. Results were presented in the form of adjusted odds ratios (AOR), and their corresponding 95% confidence intervals (CI).”

For logistic regression analysis, each of the independent variables (age, education, occupation, self-identity, alcohol consumption, number of sex acts with paying partners, frequency of receptive sex acts, number of casual partners, and knowledge of STIs) was transformed into dichotomous variables because of skewed distributions. We dichotomized age into ≤25 and 26 years or older because the average age of marriage for Indian men has been documented as 26 years. For education we created two groups – up to secondary education and above secondary education, as usually completing at least secondary education seems sufficient by many Indian families from lower socioeconomic status. Similarly occupation was categorized as agricultural/non-agricultural laborers and others including businessmen, students, in-service etc., considering the small numbers in the sub-categories and also assuming that behavior of laborers (blue collar workers) would be different from those engaged in other jobs. MSM were categorised as kothis and non-kothis (included panthis, double-decker and bisexuals), as in targeted interventions kothis are relatively highly visible and well organised, and mostly reached through interventions when compared with other subgroups (such as panthi or double-decker)[14]. For alcohol consumption, we created two groups: drinkers and non-drinkers as we did not assess the frequency of drinking using a standardized instrument. As the variables ‘number of sex acts with a male paying partner in the past week’ and ‘number of male casual partners in the past month’ were highly skewed, they were dichotomized as <5 and ≥5 and above for number of sex acts with paying partners and <7 and ≥7 and above for number of casual partners.

## Results

Of the total 1618 MSM, nearly two-thirds of respondents were 26 years or older. About four-fifths (79.4%) of the MSM self-identified as kothis, 15.3% as double-deckers and 4.0% as bisexuals. About three-fifths of MSM had above secondary level education (59.5%) and one-fourth reported having been ever married (24.5%). Almost half of the respondents reported being in debt and four-fifths reported consuming alcohol in the past month and 11.1% had ever consumed any drugs. Forty one percent of MSM reported having all three types of partners combined. Those reported having regular, paying, and casual male partners were 79.7%, 91.3% and 54.4%, respectively. Among MSM having all types of partners combined, 52.6% (n=683) reported using condoms consistently during anal sex. Consistent condom use with regular male partners was 45.3% (n=1259); the corresponding proportion with paying and casual male partners was 50.8% (n=1425) and 57.9% (n=842), respectively (Table 1).

**Table 1:** Demographic characteristics and condom use behaviour with different types of male sexual partners of MSM in Tamil Nadu, 2009-2010

**Indicators**	%* (number)	**Indicators**	%* (number)
**Socio-demographics**		**Condom use**	
**Education**		**Consistent condom use with all three partners combined**	
Illiterate	6.2 (126)	No	47.3 (226)
Up to secondary education	34.2 (492)	Yes	52.6 (457)
Above secondary	59.5 (1000)	**Consistent condom use with regular male partners**	
**Age (years)**		No	54.6 (556)
≤ 25	33.8 (597)	Yes	45.3 (703)
26–35	44.3 (728)	**Consistent condom use with paying male partners**	
36–45	17.9 (227)	No	49.1 (577)
≥46 and above	3.8 (66)	Yes	50.8 (848)
**Occupation**		**Consistent condom use with casual male partners**	
Student/unemployed	7.0 (105)	No	42.0 (250)
Self-employed/business/service (govt./pvt)	47.3 (753)	Yes	57.9 (592)
Agricultural/non-agricultural labor	37.7 (666)		
Masseur	2.0 (25)	**Sexual behavior**	
Sex worker	4.9 (51)	**Frequency of receptive sex with regular male partner**	
Others	0.88 (18)	Never/in-frequent	29.0 (293)
**Marital status**		Frequent	70.9 (956)
Never married	75.5 (1228)	**No. of times had anal intercourse in past week with paying male partner**	
Ever married	24.5 (390)	<5	66.8 (945)
**Self-identity~**		≥5 and above	33.1 (480)
Kothi	79.4 (1320)	**Other casual male partners in past month**	
Panthi	1.2 (48)	<7	70.4 (667)
DD	15.3 (194)	≥7 and above	29.5 (199)
Bisexual	4.0 (56)		
**In debt**		**Risk perception and Exposure**	
No	51.3 (866)	**Feels at risk of acquiring HIV**	
Yes	48.6 (752)	No	63.9 (1029)
		Yes	36.0 (589)
**Substance use**		**Membership in a community-based organization**	
**Alcohol consumption (past month)**		No	46.4 (785)
Everyday	9.9 (115)	Yes	53.5 (831)
At least once a week	35.4 (621)	**Knowledge of STI symptoms**	
Less than once a week	35.2 (538)	Knows <3 symptoms	12.2 (199)
Never consumed	19.3 (344)	Knows 3+ symptoms	87.7 (1419)
**Drug use**		**Exposed to any HIV prevention interventions**	
No	88.8 (1528)	No	8.2 (179)
Yes	11.1 (90)	Yes	91.7 (1439)
**Types of male sexual partners**			
**Having all three male partners combined**			
No	58.7 (935)		
Yes	41.3 (683)		
**Having regular male partner**			
No	20.2 (359)		
Yes	79.7 (1259)		
**Having paying male partner**			
No	8.6 (145)		
Yes	91.3 (1473)		
**Having casual male partner**			
No	45.5 (752)		
Yes	54.4 (866)		

*Weighted percentages; n=1618.

Note: totals may not add up in all variables because of exclusion of missing data and do not know responses.

~Kothi - feminine/receptive; Panthi - masculine/insertive; DD - Double Decker, Insertive and receptive.

Table 2 shows the results from the multiple analyses for consistent condom use with all three types of partners combined, and separately for regular, paying and causal male partners. In the controlled analysis, factors associated with increased odds of consistent condom use with all three types of partners combined were engagement in receptive anal sex on a frequent basis (adjusted odds ratio [AOR] 2.17, 95% confidence interval [CI] 1.01-4.65), fewer number (less than 7) of casual partners in the past month (AOR 3.41, 95% CI 1.50-7.73), and membership in a community-based organization for MSM (AOR 3.54, 95% CI 1.62-7.74). The factors associated with decreased odds for consistent condom use with all three types of partners combined were age 26 years or older (AOR 0.31, 95% CI 0.15-0.62), alcohol use (AOR 0.28, 95% CI 0.14-0.58), drug use (AOR 0.18, 95% CI 0.03-0.95), and being in debt (AOR 0.49, 95% CI 0.24-0.99).

**Table 2:** Factors associated with reported consistent condom use among MSM with all three types of partners combined, regular, paying, and casual male partners in Tamil Nadu, 2009-2010

	^**a**^**All three types of partners combined**	^**b**^**Regular male partners**	^**c**^**Paying male partners**	^**d**^**Casual male partners**
**Indicators**	**Adjusted OR (95% CI)**	**Adjusted OR (95% CI)**	**Adjusted OR (95% CI)**	**Adjusted OR (95% CI)**
**Socio-demographics**				
**Education**				
Up to secondary education	Referent	Referent	Referent	Referent
Above secondary	1.76 (0.92–3.37)	1.07 (0.70–1.62)	1.20 (0.81–1.79)	1.60 (0.86–2.97)
**Age**				
≤25 years	Referent	Referent	Referent	Referent
26 years or older	0.31 (0.15–0.62)^**^	0.54 (0.32–0.90)^*^	0.72 (0.46–1.13)	0.45 (0.24–0.85)^*^
**Occupation**				
Agri/non-agricultural labor	Referent	Referent	Referent	Referent
Student/unemployed/business/service/others	0.74 (0.37–1.47)	0.83 (0.53–1.30)	0.85 (0.56–1.27)	0.91 (0.48–1.71)
**Marital status**				
Never married	Referent	Referent	Referent	Referent
Ever married	2.34 (0.97–5.64)	1.13 (0.61–2.12)	0.73 (0.39–1.34)	0.93 (0.38–2.22
**Self-identity**				
Panthi/DD/bisexual	Referent	Referent	Referent	Referent
Kothi	0.85 (0.26–2.77)	0.81 (0.42–1.55)	0.66 (0.33–1.33)	0.70 (0.30–1.64)
**In debt**				
No	Referent	Referent	Referent	Referent
Yes	0.49 (0.24–0.99)^*^	0.40 (0.25–0.64) ^**^	0.54 (0.35–0.84)^**^	0.55 (0.29–1.01)
**Substance use**				
**Alcohol consumption**				
No	Referent	Referent	Referent	Referent
Yes	0.28 (0.14–0.58)^**^	0.50 (0.30–0.85) ^*^	0.35 (0.22–0.57) ^**^	0.34 (0.17–0.67) ^**^
**Drug use**				
No	Referent	Referent	Referent	Referent
Yes	0.18 (0.03–0.95)^*^	0.46 (0.16–1.31)	0.63 (0.23–1.75)	0.18 (0.05–0.65) ^**^
**Frequency of receptive sex acts, no. of sex acts and no. of partners**				
**Frequency of receptive sex with regular**				
**male partner**				
Never/in-frequent	Referent	Referent	N/A	N/A
Frequent	2.17 (1.01–4.65)^*^	1.25 (0.76–2.07)		
**No. of times had anal intercourse in past**				
**week with paying male partner**				
<5	Referent	N/A	Referent	N/A
5 and above	1.77 (0.93–3.36)		1.31 (0.83–2.08)	
**Casual male partners in past month**				
7 and above	Referent	N/A	N/A	Referent
<7	3.41 (1.50–7.73)^**^			0.90 (0.41–2.01)
**Risk perception and exposure**				
**Feels at risk of acquiring HIV**				
No	Referent	Referent	Referent	Referent
Yes	1.31 (0.64–2.67)	1.40 (0.92–2.15)	1.92 (1.22–3.01) ^**^	1.68 (0.90–3.15)
**Membership in a community-based organization (CBO)**				
No	Referent	Referent	Referent	Referent
Yes	3.54 (1.62–7.74)^**^	1.96 (1.23–3.11) ^**^	1.46 (0.94–2.27)	1.67 (0.84–3.31)
**Knowledge of STI symptoms**				
Knows <3 symptoms	Referent	Referent	Referent	Referent
Knows 3+ symptoms	1.51 (0.45–4.97)	2.00 (0.92–4.34)	1.19 (0.56–2.53)	0.77 (0.27–2.15)
**Exposed to any HIV prevention intervention**				
No	Referent	Referent	Referent	Referent
Yes	3.12 (0.48–19.91)	2.15 (0.81–5.65)	0.79 (0.35–1.77)	3.62 (1.31–10.00) ^*^

^a^‘All three types of partners combined’ here refers to having regular, paying, and casual male partners.

^b^Reported only by those who had regular male partners.

^C^Reported only by those who had paying male partners.

^d^Reported only by those who had casual male partners.

*p <.05, **p<.01.

*N/A* Not available.

With regular partners, membership in a community-based organization for MSM (AOR 1.96, 95% CI 1.23-3.11) was associated with increased odds of consistent condom use, whereas age 26 years or older (AOR 0.54, 95% CI 0.32-0.90), in debt (AOR 0.40, 95% CI 0.25-0.64) and alcohol use (AOR 0.50, 95% CI 0.30-0.85) were associated with decreased odds.

With paying partners, perceived higher risk of acquiring HIV (AOR 1.92, 95% CI 1.22-3.01 ) was associated with increased odds for consistent condom use, and being in debt (AOR 0.54, 95% CI 0.35-0.84) and alcohol use (AOR 0.35, 95% CI 0.22-0.57) were associated with decreased odds.

With casual partners, exposure to any HIV prevention intervention (AOR 3.62, 95% CI 1.31-10.0) was found to be significantly associated with consistent condom use, whereas age 26 years or older (AOR 0.45, 95% CI 0.24-0.85), alcohol use (AOR 0.34, 95% CI 0.17-0.67) and drug use (AOR 0.18, 95% CI 0.05-0.65) were associated with decreased odds. We did not find significant association of other factors such as level of education, occupation, sexual identity, and knowledge of STIs with consistent condom use with any type of partner and all three types of partners combined.

## Discussion

Our study found that only half of the MSM respondents were consistently using condoms during anal sex with their male partners. A higher proportion of MSM reported consistent condom use with casual male partners when compared with regular or paying male partners. Engagement in frequent receptive anal sex acts, having fewer casual partners, membership in a community-based organization for MSM were factors associated with increased odds for consistent condom use with MSM having all three types of partners combined. However, not all these factors were found to be significantly associated with consistent condom use when examined partner-wise. Consistent condom use with regular male partners was associated with membership in a community-based organization; consistent condom use with paying and casual male partners was associated with perceived higher risk of acquiring HIV and exposure to any HIV prevention intervention, respectively. Being aged 26 years or older, being in-debt, and alcohol use were factors associated with inconsistent condom use across partner types.

Those MSM who had receptive anal sex on a frequent basis with regular partners and who had fewer casual partners used condoms consistently with all three types of partners combined. Adequate access to both free and subsided condoms through interventions prior to and during this study [35,36] and favorable peer norms towards condom use could have contributed to consistent condom use. These findings might reflect the intention of MSM in protecting their male regular and casual partners and thus themselves from HIV infection. Being exposed to HIV prevention interventions could also have inculcated a sense of responsibility to their male regular partners, and a desire to avoid passing infection to them. However, exposure to any HIV prevention intervention was not statistically significant with condom use with different partner types (except for casual partners). This is despite free availability of condoms from the program [37]. Qualitative research could probably offer plausible explanations on how the free condoms distributed in HIV interventions are used with different types of partners and whether, and in what ways, the differences in condom use with different types of partners are associated with program exposure.

MSM who had higher self-perceived risk for HIV were more likely to use condoms with their paying male partners. This points out that the current interventions may be successful in increasing the self-risk perception in relation to paying partners, but not necessarily with other types of partners – especially regular partners. Similarly, MSM who were members of community-based organizations were more likely to use condoms with all three types of male partners or regular male partners. We hypothesize that membership in a community-based organization provided an opportunity for these MSM to interact with other MSM and collectively empowered them to resist unprotected sex with paying partner and to even understand the need to consistently use condoms with regular male partners. At least one study from the United States has documented that engagement with community agencies serving MSM are more likely to be associated with safer sex behaviors among MSM, possibly due to the social support obtained from friendship networks and other resources of the community agencies [38]. Even though there is evidence of positive impact of collectivization on safer sex practices among female sex workers [39-42], there are hardly any studies on the effects of collectivization on safer sex practices among Indian MSM [43], which need to be explored further. Available evidence suggests that interventions involving community-building empowerment activities can lead to significant risk reduction in MSM [21,44].

With any type of partners, MSM 26 years or older were found to be less likely to use condoms consistently. It may be due to the predominant focus of HIV interventions on younger MSM, and limited coverage of relatively older men (MSM) [14], who may decrease or stop visiting cruising or intervention sites once they get married[45]. Alternatively, it could be due to ‘prevention fatigue’ among older MSM [46,47], which would warrant different strategies to maintain safer sex behaviors to prevent them from relapsing into unsafe sex. Similar finding has been reported in studies from other developing countries like Bangkok [48], Africa [49] and China [50] where the older MSM were less likely to use condoms consistently, even though studies from developed countries have often reported younger MSM to engage in high risk behaviors [46,51].

A high proportion of MSM (four-fifths) in our study reported consuming alcohol and it was associated with inconsistent condom use across all partner types. Similar finding was reported from another study conducted among MSM in Chennai [52]. Concerns over the association between alcohol and drug use, sexual practices and HIV have been raised earlier as well [53-55]. It is therefore important to integrate messages on connection between alcohol use and HIV-related sexual risk within the existing HIV prevention programs among MSM, and provide referrals services if problematic alcohol use is identified among MSM.

Our study has some limitations. Our findings may be generalizable only to those MSM who are coming to cruising sites and to those MSM who engage in anal sex for cash or kind. Similarly, due to socio-cultural variations, our findings may not be generalizable to MSM in other states in India. Also, self-reported condom use may be biased due to socially desirable responses from the survey participants. In spite of these limitations, our study is first among the few large-scale studies among this hard-to-reach population that have identified possible factors associated with consistent condom use among MSM by type of male partners. Given the call for need for prevention strategies tailored to the typology of MSM [38] and type of male partners [9], findings from our analysis will be useful for policymakers and practitioners.

## Conclusion

Our study found that a significant proportion of MSM are not using condoms consistently with their male partners, and consistent condom use varied with the type of male partner – relatively less with regular partners when compared with paying or casual partners. Consistent condom use was associated with membership in a community-based organization for MSM and exposure to HIV prevention intervention. HIV interventions among MSM need to promote consistent condom use with all types (regular, paying, and causal) of male partners, and need to reach MSM from all age groups. In addition to enhancing interventions that focus on individual level risk reduction, it is important to undertake structural interventions that aim to create an enabling legal environment and promote social acceptance of same-sex sexuality, and screen for and address factors such as alcohol and drug use, and financial hardship that act as contextual barriers to consistent condom use.

## Abbreviations

MSM: Men who have sex with men; NACO: National AIDS Control Organization; STI: Sexually transmitted infections; IBBA: Integrated behavioural and biological assessment; ICMR: Indian Council of Medical Research; CCU: Consistent condom use.

## Competing interests

The authors declare that they have no competing interests.

## Authors’ contributions

SR, VC, LR and PG contributed to conception, design, writing and finalization of the manuscript. All other authors have read and approved the final manuscript.

## Pre-publication history

The pre-publication history for this paper can be accessed here:

http://www.biomedcentral.com/1471-2458/13/827/prepub
